# Genetic mapping of wild introgressions into cultivated peanut: a way toward enlarging the genetic basis of a recent allotetraploid

**DOI:** 10.1186/1471-2229-9-103

**Published:** 2009-08-03

**Authors:** Daniel Foncéka, Tossim Hodo-Abalo, Ronan Rivallan, Issa Faye, Mbaye Ndoye Sall, Ousmane Ndoye, Alessandra P Fávero, David J Bertioli, Jean-Christophe Glaszmann, Brigitte Courtois, Jean-Francois Rami

**Affiliations:** 1 Centre de coopération internationale en recherche agronomique pour le développement (Cirad), UMR Développement et Amélioration des plantes, TA A96/3, Avenue Agropolis, Montpellier, France; 2 ISRA: Institut Sénégalais de Recherches Agricoles, Centre National de Recherche Agronomique, BP 53, Bambey, Sénégal; 3 ISRA-CERAAS: Institut Sénégalais de Recherches Agricoles, Centre d'Etude Régional pour l'Amélioration de l'Adaptation à la Sécheresse, Route de Khombole, BP 3320, Thiès, Sénégal; 4 Embrapa Recursos Genéticos e Biotecnologia, C.P. 02372, CEP 70.770-900 Brasilia, DF, Brazil; 5 Universidade Católica de Brasília, Campus II, SGAN 916, CEP 70.790-160 Brasilia, DF, Brazil; 6 Universidade de Brasília, Campus Universitário, CEP 70.910-900 Brasília, DF, Brazil

## Abstract

**Background:**

Peanut (*Arachis hypogaea *L.) is widely used as a food and cash crop around the world. It is considered to be an allotetraploid (2n = 4x = 40) originated from a single hybridization event between two wild diploids. The most probable hypothesis gave *A. duranensis *as the wild donor of the A genome and *A. ipaënsis *as the wild donor of the B genome. A low level of molecular polymorphism is found in cultivated germplasm and up to date few genetic linkage maps have been published. The utilization of wild germplasm in breeding programs has received little attention due to the reproductive barriers between wild and cultivated species and to the technical difficulties encountered in making large number of crosses. We report here the development of a SSR based genetic map and the analysis of genome-wide segment introgressions into the background of a cultivated variety through the utilization of a synthetic amphidiploid between *A. duranensis *and *A. ipaënsis*.

**Results:**

Two hundred ninety eight (298) loci were mapped in 21 linkage groups (LGs), spanning a total map distance of 1843.7 cM with an average distance of 6.1 cM between adjacent markers. The level of polymorphism observed between the parent of the amphidiploid and the cultivated variety is consistent with *A. duranensis *and *A. ipaënsis *being the most probable donor of the A and B genomes respectively. The synteny analysis between the A and B genomes revealed an overall good collinearity of the homeologous LGs. The comparison with the diploid and tetraploid maps shed new light on the evolutionary forces that contributed to the divergence of the A and B genome species and raised the question of the classification of the B genome species. Structural modifications such as chromosomal segment inversions and a major translocation event prior to the tetraploidisation of the cultivated species were revealed. Marker assisted selection of BC_1_F_1 _and then BC_2_F_1 _lines carrying the desirable donor segment with the best possible return to the background of the cultivated variety provided a set of lines offering an optimal distribution of the wild introgressions.

**Conclusion:**

The genetic map developed, allowed the synteny analysis of the A and B genomes, the comparison with diploid and tetraploid maps and the analysis of the introgression segments from the wild synthetic into the background of a cultivated variety. The material we have produced in this study should facilitate the development of advanced backcross and CSSL breeding populations for the improvement of cultivated peanut.

## Background

Peanut (*Arachis hypogaea *L.) is widely used as a food and cash crop around the world. It is mainly grown by resource-poor farmers in Africa and Asia to produce edible oil, and for human and animal consumption. Peanut is a member of the *Fabaceae*, tribe *Aeschynomeneae*, subtribe *Stylosanthinae*, genus *Arachis*. In this genus, 69 diploid and tetraploid species have been described [1]. *A. hypogaea *is the only species that has been truly domesticated although several species have been cultivated for their seed or forage [2]. Cultivated peanut is considered to be an allotetraploid (2n = 4x = 40) originated from a single hybridization event between two wild diploids with A and B genome [3]. Several studies aimed at identifying the wild diploid ancestors of *A. hypogaea*. The wild species *A. duranensis *and *A. ipaënsis *appeared to be the best candidates for the A and B genome donors, respectively [4-6].

Polyploidy is a widespread process that played a major role in higher plants' speciation and adaptation. The stages of polyploid formation usually include reproductive isolation from the progenitors [7,8]. As for many polyploid species, cultivated peanut has experienced a genetic bottleneck which, superimposed with the effects of the domestication, has greatly narrowed the genetic diversity. The low level of DNA polymorphism between cultivated genotypes has been described by many authors [9-12]. More recently, a rate of polymorphism of 12.6% has been reported between two cultivated varieties, used as parents of a RIL population, surveyed with 1145 SSR markers [13]. The low level of polymorphism within cultivated peanut has greatly hampered the application of molecular breeding approaches for the genetic improvement of cultivated peanut. Up to date, few genetic linkage maps have been published in *Arachis*. At the diploid level, three genetic maps involving species with A and B genomes, one based on RFLP markers [14] and the other ones on SSR markers [15,16], have been produced. The A genome SSR based map has been recently extended using legume anchor markers and aligned with *Medicago *and *Lotus *genomic sequences [17]. At the tetraploid level, two genetic maps were also reported. Varshney et al. [13] reported the detection of drought tolerance QTLs based on a cultivated × cultivated SSR genetic map. Although the genetic map remained unsaturated, due to the low level of polymorphism between cultivated peanut varieties, QTLs have been detected attesting of the interest of molecular breeding tools in genetic improvement of peanut. Burow et al. [18] reported the construction of a RFLP map, based on a BC_1 _population deriving from a cross between a wild synthetic amphidiploid (TxAG6) and a cultivated peanut variety (Florunner). The synthetic amphidiploid, used to overcome the reproductive barriers between the wild diploids and the cultivated species, allowed the genome-wide analysis of the transmission of chromatin between wild and cultivated species of the genus *Arachis*. However, the wild parents used to create the amphidiploid (*A. batizocoi, A. cardenasii *and *A. diogoii*) are unlikely to be the ancestors of *A. hypogaea *[12,19-21]. The genetic mapping of populations derived from the cross between the most probable wild progenitors of *A. hypogaea *and a cultivated peanut variety has, to our knowledge, never been reported.

Genome-wide introgression of a small fraction of the wild genome species while keeping the genetic background of the cultivated is a good mean to explore the largely untapped reservoir of useful alleles of interest that remain in the wild species. This is especially interesting for species with narrow genetic basis. This approach has been widely utilized for the introgression of favourable QTL(s) for various traits in tomato [22-26], in rice [27-32], in wheat [33] and in barley [34,35]. In peanut, the reproductive barriers between wild and cultivated species, the technical difficulties encountered in making large number of crosses as well as the short period between sowing and flowering have impeded the efforts to apply a Marker Assisted Backcross (MABC) approach for the development of interspecific introgression line populations.

In this study, we report for the first time the development and the analysis of the genome-wide segment introgressions of the most probable wild progenitors of the cultivated peanut species (*A. duranensis *and *A. ipaënsis*) into the background of the cultivated Fleur 11 variety through the construction of a SSR genetic map as well as the evaluation of the coverage and the length of the wild genome segments in a BC_1_F_1 _and BC_2_F_1 _populations. This work benefits from the recently developed synthetic amphidiploid (*A. ipaënsis *× *A. duranensis*)^4X ^[5] that made possible the interspecific introgressions.

## Methods

### Plant material

**Figure 1:**
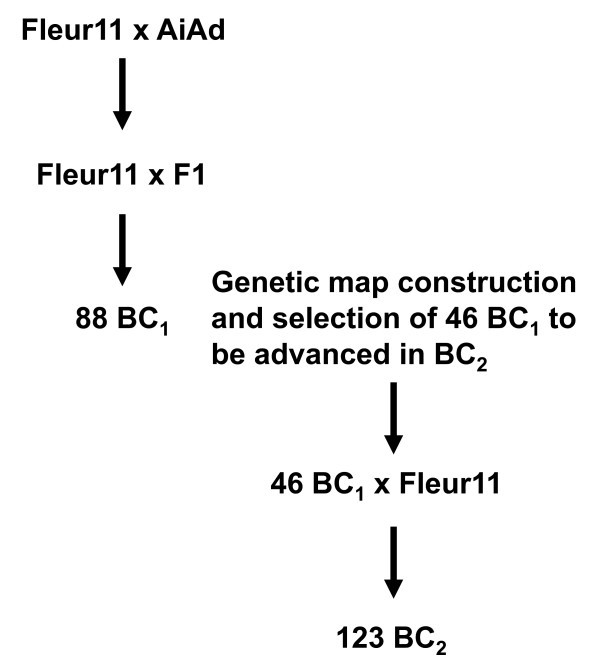
**Breeding scheme used in the study**. The cultivated Fleur 11 variety was used as female parent to produce the F_1 _and the BC_1_F_1 _individuals, and as male parent for producing the BC_2_F_1 _individuals.

A panel comprising 2 wild diploid accessions (*A. duranensis *V14167 diploid AA and *A. ipaënsis *KG30076 diploid BB), a tetraploid AABB amphidiploid (*A. ipaënsis *× *A. duranensis*)^4X^, hereafter called AiAd and a cultivated tetraploid AABB variety (Fleur 11), was used in this study. The amphidiploid was developed by Favero et al. [5] by crossing *A. ipaënsis *KG30076 (B genome) with *A. duranensis *V14167 (A genome). The resulting F_1 _was doubled with colchicine to produce a fertile fixed synthetic amphidiploid. Fleur 11, a local peanut variety grown in Senegal, is a Spanish type short cycle variety, high yielding and tolerant to drought. A BC_1_F_1 _and a BC_2_F_1 _populations deriving from the cross between Fleur 11 used as female recurrent parent and the amphidiploid AiAd were produced. The BC_1_F_1 _and BC_2_F_1 _populations were developed under greenhouse conditions in Senegal in 2006 and 2008 respectively. The crossing scheme used to generate the two populations is shown in Figure 1. The BC_1_F_1 _population comprised 88 individuals. Forty six BC_1_F_1 _plants were selected based on introgression analysis and crossed with the Fleur 11 recurrent parent to produce the BC_2_F_1 _generation.

### DNA Isolation

Young leaves were harvested from 15 day old plants and immediately stored at 4°C in ice before DNA extraction. DNA was extracted from 100 mg of fresh leaves following a slightly modified MATAB protocol [36]. Briefly, leaves were ground in liquid nitrogen using a mortal and pestle and dissolved in 750 μL of MATAB buffer at 74°C. The samples were incubated 20 minutes at 74°C and cooled during 5 minutes at room temperature. A volume of 750 μL of CIA (24:1) was added in each sample and all samples were shaken gently until homogenization before centrifugation at 12000 rpm during 20 minutes. The supernatant was harvested and the DNA was precipitated with 600 μL of 2-propanol. After centrifugation, pellets were washed with 300 μL of 70% ethanol, air dried and dissolved in 500 μL of TE.

### Microsatellite Analysis

Four hundred twenty three already-published SSR markers [12,15,21,37-45] plus 135 unpublished long size SSR markers from EMBRAPA and the Universidade Católica de Brasília were used in this study. A total of 558 SSR markers have been screened for polymorphism on the amphidiploid and its two wild diploid parents, and on the cultivated Fleur 11 variety. For a given SSR locus, the forward primer was designed with a 5'-end M13 tail (5'-CACGACGTTGTAAAACGAC-3'). PCR amplifications were performed in a MJ Research PTC-100™ thermocycler (Waltham, MA, USA) or in an Eppendorf Mastercycler on 25 ng of DNA in a 10 μl final volume of buffer (10 mM Tris-HCl (pH 8), 100 mM KCl, 0.05% w/v gelatin, and 2.0 mM MgCl2) containing 0.1 μM of the M13-tailed primer, 0.1 μM of the other primer, 160 μM of dNTP, 1 U of Taq DNA polymerase (Life Technologies, USA.) and 0.1 μM of M13 primer-fluorescent dye IR700 or IR800 (MWG, Germany). The touchdown PCR programme used was as follow: initial denaturation at 95°C for 1 min; following by 10 cycles of 94°C for 30 s, Tm (+5°C, -0.5°C/cycle) for 1 min, and 72°C for 1 min. After these cycles, an additional round of 25 cycles of 94°C for 30 s, Tm for 1 min, and 72°C for 1 mn and a final elongation step at 72°C for 8 min was performed. IR700 or IR800-labeled PCR products were diluted 7-fold and 5-fold respectively, subjected to electrophoresis in a 6.5% polyacrylamide gel and then sized by the IR fluorescence scanning system of the sequencer (LI-COR, USA). Migration images were analysed using Jelly 0.1 (Rami, unpublished) and exported as a data table. Segregations were checked for distortion to the expected 1:1 ratio using a Chi^2 ^test at a significance level of 0.05.

### Genetic map construction

**Figure 2:**
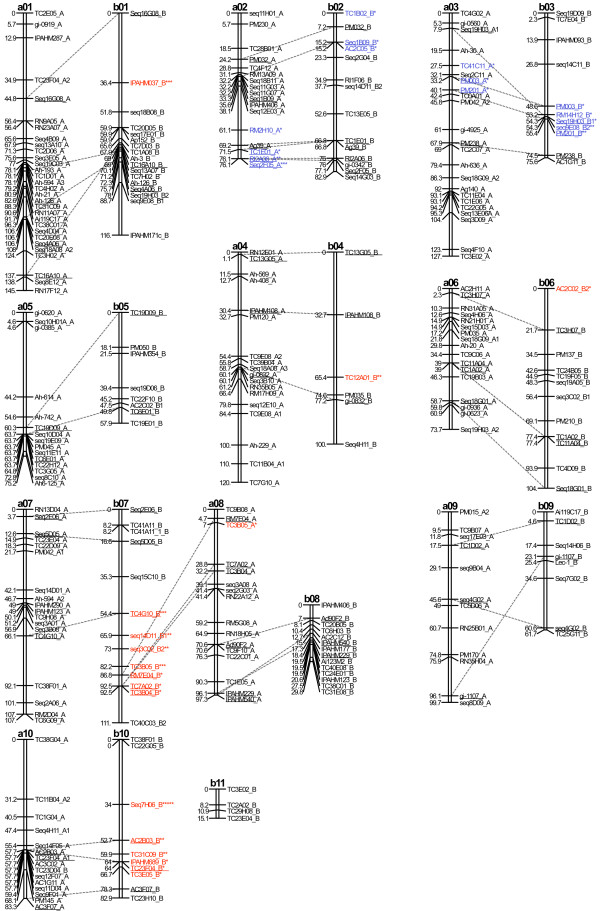
**Genetic map and synteny between the A and the B genomes**. The LGs deriving from the A genome are named from a01 to a10 and those deriving from the B genome from b01 to b11. Map distances are given in Kosambi centimorgans. Common markers between pair of homeologous LGs are underlined and connected with dashed lines. Markers placed at LOD < 4 are represented in italics, and those that amplified more than one locus on the same genome are identified by the number 1, 2 and 3. Loci showing significant segregation distortion (P < 0.05) are identified by stars following locus name. The colour and number of stars specify the direction and the intensity of the segregation distortion respectively. Blue: markers skewed toward the alleles of the cultivated parent. Red: markers skewed toward the alleles of the wild parent.

The polymorphic markers were used to genotype 88 individuals of the BC_1_F_1 _population. The linkage analysis was performed using Mapdisto software version 1.7.2.4 [46] and CarthaGene software version 1.0 [47]. The origins of the alleles (A or B genomes) were determined by comparison to the alleles coming from the diploid progenitors of the amphidiploid. Mapdisto software was used in a first step, for the linkage group determination and marker ordering within each linkage group. A minimum LOD of 4 and maximum recombination fraction of 0.3 were fixed for the linkage group determination using the "find groups" command. The order of the markers within each linkage group was estimated using the "order" command. The markers that had not been placed at LOD 4 were tried at decreasing LOD, down to a LOD of 2 and a maximum recombination fraction of 0.3. These markers are indicated in italic on the map (Figure 2). The quality of the genotyping data at a specific marker was controlled using the "drop locus" command. The few markers having bad quality genotyping data were discarded from the linkage analysis. In a second step, CarthaGene software was used for the optimization of the best marker order determined by Mapdisto. This was done applying the simulated "annealing" and "greedy" algorithms. The best maps obtained were improved using the "Flips" and the "Polish" commands. Genetic distances between markers were computed using Kosambi mapping function.

### Introgression analysis

From the map of 298 SSR markers previously developed on the BC_1_F_1 _generation, a framework map comprising 115 SSR markers was derived. Compared to the initial map, this framework offered a regular coverage of all the linkage groups. These 115 SSR markers were used to genotype 123 BC_2_F_1 _individuals.

Introgression analysis of the BC_1_F_1 _and BC_2_F_1 _populations was performed using the CSSL Finder software version 0.8b4 [48]. To select a subset of BC_1_F_1 _and BC_2_F_1 _lines providing an optimal coverage of donor genome into the recurrent background, we imposed a target length of the introgressed wild segments of 20 cM, an overlapping of adjacent segments for a given LG and the best possible return to the background of the cultivated variety.

The percentage of wild genome in the BC_1_F_1 _and BC_2_F_1 _generations and its relative diminution between the two generations, the mean size of wild introgression segments per LG and per generation, as well as the distribution of the wild segment lengths were estimated using the genotyping data available for each generation. The analysis was conducted on LGs longer than 75 cM. The lengths of the introgressed segments were calculated as the sum of consecutive intervals having a heterozygous genotype plus half the size of each flanking interval having a recurrent homozygous genotype.

## Results

### SSR polymorphism and origin of the markers

Among the 558 SSR markers screened, 333 (59.6%) were polymorphic between Fleur 11 and AiAd. At a given SSR locus, the sub-genomic origin of the alleles was determined by comparison with the alleles of the diploid parents of the amphidiploid *A. ipaënsis *and *A. duranensis *that were included on each gel. This allowed distinguishing three categories of markers among the 333 polymorphic markers: 174 SSRs that were polymorphic for the A genome (52.0%), 77 SSRs that were polymorphic for the B genome (23.0%) and 82 SSRs that were polymorphic for the two genomes (24.5%). The largest proportion of polymorphic markers originated from the A genome donor *A. duranensis *(76.6%), the B genome donor *A. ipaënsis *generating 47.6% of polymorphic markers.

### Genetic map construction

Among the 333 polymorphic SSRs, we randomly selected 118 markers polymorphic for the A genome, all the markers polymorphic the B genome and those polymorphic for the two genomes. A total of 277 SSRs were used to genotype the population of 88 BC_1_F_1 _individuals. The 232 SSR markers that showed a clear electrophoretic profile amplified 322 loci. Finally, 298 loci were mapped in 21 linkage groups (LGs), spanning a total map distance of 1843.7 cM with an average distance of 6.1 cM between adjacent markers (Figure 2). The difference of polymorphism between the A and B genomes had an effect on the number of markers mapped on each genome, and the number and size of the linkage groups. For the A genome, 181 loci were mapped in 10 LGs with a number of markers per LGs varying between 12 and 30 (average of 18.1), and the length of the LGs ranging from 73.7 cM to 145.2 cM (average of 100.5 cM). For the B genome, 117 loci were mapped on 11 LGs with a number of markers per LGs varying between 4 and 17 (average of 10.7) and the length of the LGs ranging from 15.1 cM to 111.6 cM (average of 76.2 cM).

The comparison of the A and B genomes was undertaken using 53 SSR markers that mapped on both A and B LGs. The A and B LGs were considered to be homeologous when they shared at least 2 common markers. This allowed distinguishing 8 pairs of homeologous LGs (a01/b01, a02/b02, a03/b03, a04/b04, a05/b05, a06/b06, a09/b09 and a10/b10) and one quadruplet involving the LGs a07, b07, a08 and b08. LG a07 shared three markers with the upper part of LG b07 corresponding to at least the half of this LG. The lower part of LG b07 shared three markers with the upper part of LG a08. Furthermore, the lower part of LG a08 shared three markers with LG b08 (Figure 2). The small LG b11 shared 1 marker with LG a03. An overall good collinearity was observed between homeologous LGs. However, three inversions of chromosomal segments were observed on the homeologous LGs a01/b01, a03/b03, and a09/b09. Small inversions were also observed on the homeologous LGs a08/b08. These inversions might result from artefacts as they concerned closely linked markers with more than one possible order having similar LOD values. No mosaic composition of linkage groups, where A genome markers would map together with B genome markers, was observed.

A total of 32 SSR markers (10.7%) showed significant segregation distortion at P < 0.05. Apart from 4 markers mapped on LGs b01 (IPAHM037), b04 (TC12A01), b06 (AC2C02), and LG a08 (TC3B05) all the distorted markers were concentrated in specific zones of 6 different LGs (a02, b02, a03, b03, b07 and b10). Differences between the A and B genomes were also observed. For the A genome, only 8 markers (4.4%) showed segregation distortions compared to 24 (20.3%) for the B genome. For the A genome, in the zones of distortion of LGs a02 and a03, all the distorted markers were skewed toward the alleles of the cultivated parent. For the B genome, the zones of distorted markers of LGs b02 and b07 were skewed toward the allele of the wild parent while those of b03 and b10 were skewed toward the allele of the cultivated parent.

Fifteen primer pairs (AC2C02, Ah-594, PM042, Seq14D11, Seq18A03, Seq18G09, Seq19H03, Seq3C02, Seq4H11, Seq9E08, TC11B04, TC9E08, TC19E01, TC23F04 and TC40C03) amplified consistently more than one locus on the same genome. We were able to map the duplicated loci for the markers Ah-594, PM042, Seq18A03, Seq18G09, Seq19H03, TC11B04 and TC9E08. Apart from the loci amplified by TC9E08 that mapped on the same LG (a04), the loci amplified by AC2C02, Ah-594, PM042, Seq14D11, Seq18A03, Seq18G09, Seq19H03, Seq3C02, Seq4H11, Seq9E08, TC11B04, TC19E01, TC23F04 and TC40C03 mapped on different LGs suggesting possible segmental duplications. These markers were identified by the number 1, 2 and 3 on the map (Figure 2).

### Comparison with peanut published genetic maps

#### Conserved structural features between tetraploid maps

The present tetraploid map was compared to the RFLP based tetraploid BC_1_F_1 _map published by Burow et al. [18], further called "Burow's map" involving a cross between *A. hypogaea *variety Florunner and the synthetic amphidiploid TxAG6 ([(*A. batizocoi *× (*A. cardenasii *× *A. diogoii*)]^4X^). *A. batizocoi *was considered to be the B genome donor and *A. cardenasii *and *A diogoi *were the donors of the A genome. In that cross, 23 LGs spanning a total genetic distance of 2210 cM (Kosambi mapping function) were obtained. This map size was slightly larger than our map. A similar number of loci had been mapped on the A genome (156 for Burow's map versus 181 for our map) but the number of loci mapped on the B genome of the Burow's map was about 2 fold larger than on the present map (206 versus 117 respectively). The mean length of the A genome LGs was similar between the 2 maps (93.7 cM for the Burow's map vs 100.5 cM for our map) while the mean length of the B genome LGs of the Burow's map was 1.2 larger than that of the present map (94.1 cM for the Burow's map versus 76.2 cM for our map). The difference in map size between the two studies seems related to the difference of the number of mapped markers on the B genome. Interestingly, conservation of synteny between one B genome LG and two A genome LGs was observed in the two maps. On our map, LG b07 shared common markers with LG a07 and a08 while on Burow's map, LG 19 shared common markers with LG 9.1 and 9.2. Moreover, Burow et al. [18] has reported structural differences, mainly chromosome segment inversion, between four pairs of homeologous LGs (LG1/LG11, LG7/LG17, LG4/LG14 and LG5/LG15). Inversions of chromosomal segments have been observed for at least 3 LGs in our map (a01/b01, a03/b03 and a09/b09).

#### Comparison with diploid map

The results from the present tetraploid map were compared to the SSR based diploid F_2 _map [15], involving a cross between two wild diploids with A genome, *A. duranensis and A. stenosperma*. In that population, 11 LGs covering a total map length of 1230.8 cM (Kosambi mapping function) have been described. The total map length was slightly longer than what we obtained in our map when considering the total size of the LGs of the A genome (1005.2 cM). The proportion of distorted markers found in the study of Moretzsohn et al. [15] was higher than what we recorded for our A genome map (50% versus 4%). Given that a similar number of individuals were used for the map construction in the two studies, the length difference between the 2 maps might be related to the higher proportion of distorted markers on the Moretzsohn's map.

**Figure 3:**
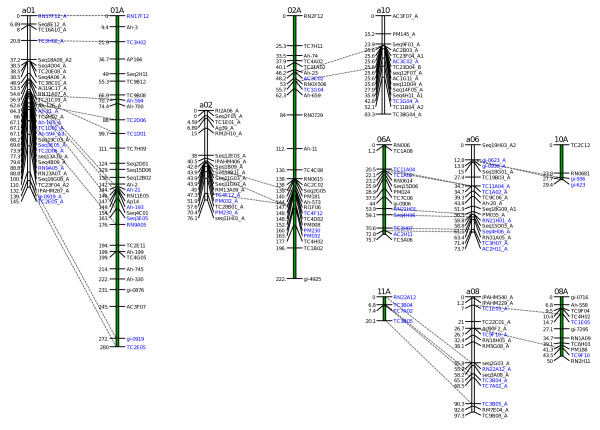
**Salient features of the comparison between the A genome LGs of our tetraploid BC1 map and the diploid AA map published by Moretzsohn et al. (2005)**. The LGs from this study are named a01, a02, a10, a06, and a08. The LGs of Moretzohn's map (01A, 02A, 06A, 10A, 08A and 11A) are represented by a green bar. Common markers between corresponding LGs in the two maps are indicated in blue, underlined and connected with dashed lines. In the two maps the distances are given in Kosambi centimorgans.

The synteny between the 2 maps was assessed with 57 common SSR markers. For all the 10 LGs of the A genome of our map, we could identify corresponding LGs in the diploid map with an overall good collinearity. The salient features of the comparison of the two maps are shown in Figure 3. The number of common SSR markers per homologous LGs varied between 2 and 11. However the synteny was not conserved for four LGs of our map when compared to the diploid map. LG a02 and a10 of our map shared 3 (PM230, PM032 and TC4F12) and 2 (AC3C02 and TC1G04) markers with LG 2 of the diploid map respectively. LGs 8 and 11 of the diploid map shared 2 (TC1E05, TC9F10) and 4 (RN22A12, TC3B04, TC7A02 and TC3B05) markers with LG a08 of our map respectively. Moreover, LG a06 of our map that was homologous to LG 6 of the diploid map shared also 2 common markers (gi-936 and gi-623) with LG 10 of the diploid map. For LGs a06 and a08 of our map, there was no evidence of spurious linkage between two different LGs as all the markers in these LGs were mapped at LOD ≥ 4.

### Introgression analysis

**Figure 4:**
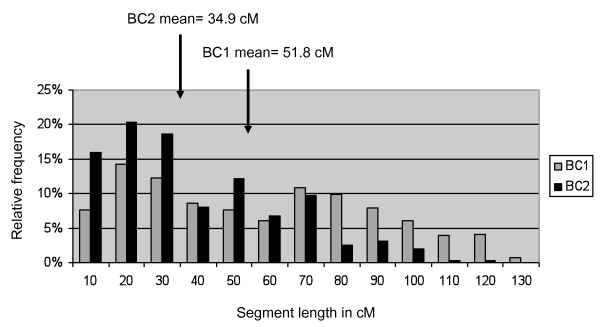
**Distribution of donor segment lengths as calculated for both the BC_1_F_1 _and the BC_2_F_2 _generations derived from the cross between Fleur 11 cultivated variety and the synthetic amphidiploid AiAd**.

In the BC_1_F_1 _generation, the percentage of heterozygous genome varied between 26.5% and 77.0% (average of 49.8%) while in the BC_2_F_1_generation it varied between 6.1% and 44.4% (average of 22.2%). This percentage is slightly inferior to the expected 25%, which is consistent with the selection that occurred at each generation for the best possible return to the background of the cultivated variety. From BC_1_F_1 _to BC_2_F_1_, we noted more than 50% reduction of the wild allele contribution to the genotypic constitution of the BC_2_F_1 _individuals. The distribution of the lengths of the wild segments in the BC_1_F_1 _and the BC_2_F_1 _generations was calculated for 14 LGs having a length comprised between 75 and 145.2 cM (Figure 4). The average lengths of the wild introgressed segments into the background of the cultivated were 51.8 cM in BC_1_F_1 _and 34.9 cM in BC_2_F_1_. From BC_1_F_1 _to BC_2_F_1 _generations, the segment lengths decreased of 33%. As shown in Figure 4, more than 15% of the BC_1_F_1 _lines and 20% of the BC_2_F_1 _lines had segment lengths comprised between 20 and 30 cM.

**Figure 5:**
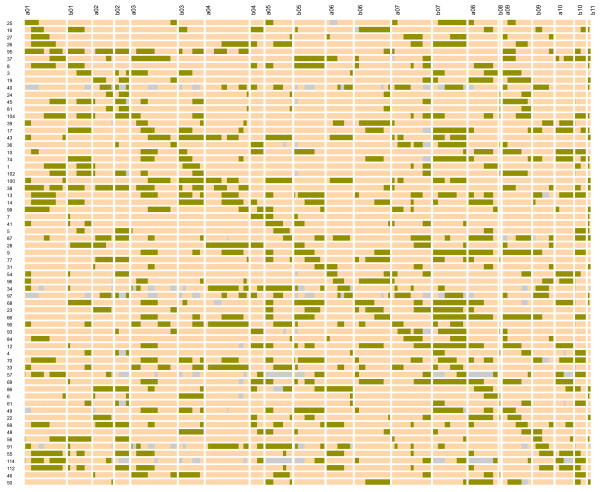
**Graphical genotype of the selected BC_2_F_1 _lines**. Each row represented a candidate line and each column a Linkage Group. The green colour indicates the heterozygous (wild/cultivated) segments and the orange colour the homozygous regions for cultivated alleles. The gray colour indicates missing data.

The CSSL-Finder software was used to select a subset of BC_1_F_1 _lines and, then, of BC_2_F_1 _lines, which ensured, in each generation, an optimal coverage of the wild genome with overlapping target segment lengths of 20 cM between neighbouring lines and the best possible return to the cultivated background. In the BC_1_F_1 _population, a subset of 22 lines was selected. The segment lengths ranged between 2.3 cM and 89.4 cM (mean of 34.8 cM). All the adjacent segments were in overlapping position and the genome percentage of the recurrent cultivated variety ranged between 38% and 68% (mean 52%). In the BC_2_F_1 _population, a subset of 59 lines was selected. The segment lengths ranged between 2.3 cM and 46.9 cM (mean of 24.5 cM) and the percentage of the recurrent background between 62% and 94% (mean of 79%). A graphical representation of the BC_2_F_1 _selected lines is shown in Figure 5. The level of coverage of the wild introgressed segments in the background of the cultivated variety was optimal both in the BC_1_F_1 _and BC_2_F_1 _populations.

## Discussion

In this study, the construction of a tetraploid molecular genetic map using a BC_1_F_1 _population and the development of a BC_2_F_1 _population allowed the analysis of the introgression of wild alleles in the background of a cultivated peanut variety. Several points have been highlighted including (1) the low level of polymorphism of the SSR markers especially between the B wild genome *A. ipaënsis *and the B genome of the cultivated, (2) the collinearity between the A and B genomes, the synteny between tetraploid and diploid maps, and the similarity between tetraploid maps, (3) the good level of introgression of the wild genome segments in the background of the cultivated variety.

### SSR polymorphism data is consistent with *A. duranensis *and *A. ipaënsis *being the most probable progenitors of the cultivated species

Cultivated peanut *Arachis hypogaea *is considered to be an allotetraploid (2n = 4x = 40) originated from a single hybridization event between two wild diploids with A and B genomes [3], followed by spontaneous duplication of the chromosomes. The identification of the wild progenitors of the cultivated peanut has been the object of numerous investigations using various approaches including cross-compatibility [5], molecular markers [4,12,20,21,49,50], biogeography [51], gene sequence comparison [52], physical mapping of rRNA genes [53] and Genome *In Situ *Hybridization (GISH) [6]. The most probable hypothesis gave *A. duranensis *as the wild donor of the A genome and *A. ipaënsis *as the wild donor of the B genome. In our study, a close relationship has been observed between the putative wild progenitor's *A. duranensis *and *A. ipaënsis *and the cultivated *A. hypogaea *var. Fleur 11 based on 558 SSR markers. *A. duranensis *and *A. ipaënsis *shared 54.1% and 72.6% of common SSR alleles with the A genome and the B genome of *A hypogaea *respectively. Moreover, 59.8% polymorphism was observed between the synthetic amphidiploid AiAd and the cultivated Fleur 11 variety. This is lower than the 83% of polymorphism that has been observed between the synthetic polyploid TxAG6 ([(*A. batizocoi *× (*A. cardenasii *× *A. diogoii*)]^4X^) and the cultivated Florunner variety based on RFLP markers [18]. This result indicates that *A. duranensis *and *A. ipaiensis *are more closely related to the A and the B genomes of the cultivated species than are *A. cardenasii *and *A. diogoi *for the A and *A. batizocoi *for the B genomes respectively. Moreover, the BC_1 _tetraploid map obtained by crossing the synthetic amphidiploid AiAd and Fleur 11 indicated a disomic inheritance of all loci. For all the LGs obtained, the markers that were polymorphic for the A genome mapped on A LGs and those polymorphic for the B genome mapped on B LGs. The chromosome pairing seems to happen between "homologous genome" attesting the high affinity between *A. duranensis *and the A genome of the cultivated species, and between *A. ipaënsis *and the B genome of the cultivated species. The same results have also been reported by Seijo et al. [6]. Our data fit well with the earlier reports indicating *A. duranensis *and *A. ipaënsis *as the most probable diploid progenitors of the cultivated peanut.

### Genome rearrangements

In this study, the synteny analysis between the A and B genomes revealed inversion of chromosomal segments for at least three LGs, and a particular feature of synteny involving the LGs a07, b07, a08 and b08 (Figure 2). Conservation of synteny between the upper region of LGs a07 and b07, and between LG b08 and the lower region of LG a08 has been pointed out. Furthermore, the upper region of LG a08 shared also three markers with the lower region of LG a07 while LG b08 lacked a large chromosomal segment that could correspond to the region of conserved synteny between LGs b07 and a08. These observations are consistent with a major translocation event that has occurred between LGs b07 and b08. Similar feature of synteny conservation between two LGs of the B genome and two LGs of the A genome have also been reported at the diploid level when comparing the *A. duranensis *× *A. stenosperma *diploid AA map [15] and the *A. ipaënsis *× *A. magna *diploid BB map [16]. Interestingly, the quadruplet of syntenic LGs in the diploid maps was also found to be syntenic with those in our map (data not shown). These observations suggest that the rearrangement between LGs b07 and b08 is an ancient translocation event that happened prior to the tetraploidisation of the cultivated peanut.

Chromosome rearrangements, including the inversion of chromosomal segments within pairs of homeologous linkage groups and the conservation of synteny between a triplet of LGs (one LG of the B genome sharing common markers with two LGs of the A genome) have also been reported by Burow et al. [18]. We were not able to identify which LGs of our map are in synteny with those of the Burow's map due to the difference of the marker types used in the two studies.

The similarity of the rearrangement events observed in the diploid and the tetraploid maps, which involve different species for A and B genomes, suggests that these evolutionary mechanisms have contributed to the divergence of the A and B genomes of the section *Arachis*. It also raises the question of the classification of the B genome species. The relationships between species with B genome remain controversial. Using RFLP makers, Gimenes et al. [44] reported a clustering of *A. batizocoi *and *A. magna *which were less related to *A. ipaënsis*, while with SSR markers, Moretzsohn et al. [21] reported a clustering of the species with B genome including *A. batizocoi*, *A. magna *and *A. ipaënsis*. Seijo et al. [6] reported, based on GISH, the distinction of *A. batizocoi *from the other B genome species and concluded that species with B genome do not seem to constitute a natural group.

The results obtained from the comparison of the diploid and tetraploid maps suggest that, based on the similarities of the rearrangement event, the species with B genome *A. ipaënsis*, *A. magna *and *A. batizocoi *could have derived from a common B genome ancestor and could be more closely related than what was previously reported based on molecular makers and on GISH. The construction of a consensus molecular genetic map involving the available diploid AA and BB maps and the tetraploid AABB maps as well as the study of crossability between species with B genome should shed new light on this issue.

Modifications of parental diploid genome following polyploidization, have been reported (for review see, [54,55]). The modifications include structural rearrangements, transposable element activation, difference in gene expression and epigenetic changes. These changes were observed in old polyploid [56-59] as well as in newly synthesized amphidiploids [60-62]. Rapid and dynamic changes in genome structure, including non additive inheritance of genomic fragments and genome-specific sequence deletion have been described in some taxa including *Brassica *[61] and wheat synthetic allotetraploids [63], but not in others including cotton [64] and sugarcane [65]. In peanut, Burow et al. [18] reported a possible genomic restructuring in the synthetic amphidiploid TxAG6 characterized by 5% of mapped alleles having unknown parental origins. In our study, we utilized a synthetic amphidiploid which had undergone several cycles of self-pollination before crossing with the cultivated allotetraploid. However, to the level of resolution afforded by our experiment, no change in genome structure has been pointed out. Further studies are needed to confirm the effectiveness and the level of genomic restructuring in peanut synthetic allotetraploid.

### Wild segment introgressions and perspectives for the development of interspecific breeding populations

Few studies have been reported in the literature regarding the genetic mapping of introgressions from wild to the cultivated peanut. Apart from the study of Burow et al. [18], introgression mapping of wild segments in the background of a cultivated variety has been reported in 46 introgression lines originated from the hybridization between *A. cardenasii *× *A. hypogaea *[66]. Considering all the lines together, introgressed segments could be found on 10 of the 11 LGs of the *A. stenosperma *× *A. cardenasii *diploid AA map [14], and represented 30% of the diploid peanut genome. The mapping of a wild segment from *A. cardenasii *conferring resistance to root-knot nematode [67] and the registration of two varieties of peanut 'COAN' [68] and 'NemaTAM' [69], having identifiable alleles conferring resistance to root-knot nematode transferred from wild species, have also been reported.

In our study, we used a synthetic wild amphidiploid as a mean for the introgression of alien alleles in the genetic background of a cultivated variety and, consequently, enlarging the genetic basis of the cultivated peanut. Genetic mapping of the wild introgressed segments gave a clear picture of the amount and the level of coverage of the wild donor genome in the background of the cultivated, and of the segment lengths and their relative decrease from BC_1_F_1 _to BC_2_F_1 _generation. The mean length of the wild segments was 51.8 cM in BC_1_F_1 _and 34.8 cM in BC_2_F_1_, and the decrease of segment size from BC_1_F_1 _to BC_2_F_1 _was about -33%. These values were similar to what was obtained in *Lycopersicon *wild × cultivated backcross populations [23]. Marker assisted selection of BC_1_F_1 _and then BC_2_F_1 _lines carrying the desirable donor segment with the best possible return to the background of the cultivated variety allowed the selection of a limited set of lines that offer an optimal coverage of the wild genome with an overlapping regions between neighbouring lines and an average segment lengths of 34.8 cM in BC_1_F_1 _and 24.5 cM in BC_2_F_1_, as well as a 79% return to the background of the cultivated variety in BC_2_F_1_. The rapid decrease of wild segment lengths observed between the BC_1_F_1 _and BC_2_F_1 _generations as well as the good level of recovery of the genetic background of the cultivated variety in BC_2_F_1 _generation is of great interest for the genetic mapping of QTLs and the development of Introgression Line (IL) libraries. ILs carrying small wild segments in a constant cultivated genetic background have the advantages of reducing epistatic and linkage drag effects and of improving the resolution of QTL mapping [23,70]. Furthermore, ILs are reliable and stable genetic resources that can be multiplied and evaluated in various environments. Many valuable sources of resistance to biotic stresses including resistance to *Cercospora *leafspot [71], to root-Knot nematode [67,72], to Peanut Bud Necrosis virus (PBNV) [73], to late leaf spot disease [74] and sources of tolerance to abiotic stresses including tolerance to thermal stress [75] and to drought (Soraya Bertioli, Vincent Vadez personal communication) were identified in peanut wild relatives. These sources can be used for genetic improvement in peanut.

The BC_1_F_1 _and BC_2_F_1 _populations that we developed are excellent starting points for the development of new breeding populations such as Advanced Backcross (AB) and Chromosome Segment Substitution Lines (CSSL) populations for analysis of the wild alleles contribution to the improvement of cultivated peanut varieties.

## Conclusion

In this study, a nearly saturated genetic map has been developed from a cross between the synthetic amphidiploid AiAd and the cultivated Fleur 11 variety. This allowed the synteny analysis of the A and B genomes, the comparison with diploid and tetraploid maps and the analysis of the introgression segments from the most probable wild progenitors of the cultivated peanut into the background of the cultivated Fleur 11 variety. The results of this study confirmed the close relationship between the wild diploids *A. duranensis*, *A. ipaënsis *and the cultivated peanut and highlighted structural rearrangements, such as chromosomal segment inversions and a major translocation event, between the A and B genome species. Finally, we showed that the low level of polymorphism reported between cultivated peanut can be overcome by using the wild species. The material we have produced in this study should facilitate the development of AB and CSSL breeding populations for the identification and utilization of valuable genes from the largely untapped reservoir of useful alleles that remained in the wild peanut species.

## Authors' contributions

DF designed and coordinated the study, was involved in genotyping data production, carried out data analyses and map construction and drafted the manuscript. HAT, IF and ON carried out crosses and population development. RR and MNS were involved in DNA extraction and genotyping data production. APF produced the synthetic amphidiploid AiAd. DJB and JCG were involved in the design of the study. BC contributed to editing of the manuscript and helped in data analysis. JFR conceived, designed and coordinated the study, was involved in map construction, and editing of the manuscript.
